# Task constraints and stepping movement of fast-pitch softball hitting

**DOI:** 10.1371/journal.pone.0212997

**Published:** 2019-02-26

**Authors:** Ryota Takamido, Keiko Yokoyama, Yuji Yamamoto

**Affiliations:** 1 Graduate School of Education and Human Development, Nagoya University, Furo-cho, Chikusa, Nagoya, Japan; 2 Research Center of Health, Physical Fitness and Sports, Nagoya University, Furo-cho, Chikusa, Nagoya, Japan; 3 Department of Psychology and Human Developmental Sciences, Nagoya University, Furo-cho, Chikusa, Nagoya, Japan; Arizona State University & Santa Fe Institute, UNITED STATES

## Abstract

This study aims to clarify the relationship between task constraints and the preparatory movement of fast-pitch softball batters in Japan for three different competition categories, namely high school, college, and league. As task constraints, we focused on the temporal and tool constraints and evaluated preparatory movements using initiation time and step duration of the stepping movement in a striking action. First, we confirmed the temporal constraints in each category and then examined the relationship between the temporal constraints and the stepping movements. The results demonstrated that the temporal constraints affected both initiation time and step duration of the stepping movements. Hierarchical cluster analysis was applied to the two variables. Consequently, the stepping movement of the softball players was classified into three types: “late initiation–short duration” (LS), “early initiation–short duration” (ES), and “early initiation–long duration” (EL) corresponding to the task constraints. Finally, the relationship between the task constraints of each category and the stepping movements was examined. The results revealed that high school players exhibited mostly the LS- or EL-type stepping movement; college players exhibited mostly the EL-type stepping movement; and league players exhibited all types. These results depict that the players in each category with each temporal and tool constraint exhibited particular types of stepping movements as a preparatory movement corresponding to each task constraint, thereby arguing that players use a not-to-lose strategy exploiting the redundancy in solutions under various task constraints.

## Introduction

We can perform a task-oriented behavior corresponding to the changes of environmental demands. A typical example is an interpersonal and/or team sports activity. In martial arts, experts perform attacking and defending maneuvers based on the not-to-lose strategy, adjusting optimal interpersonal distance [1–4]. In team sports, experts can perceive the movements of other teammates and coordinate with them [5, 6]. These complex movements are performed under various task constraints, such as rules or using tools. Players, especially in interpersonal and/or team sports, are required to undertake quick decision-making and provide an accurate response. Accordingly, temporal constraints have become more important.

In striking sports, such as tennis, squash, and bat-and-ball games like cricket and baseball, players are required to accurately predict the ball speed and direction, that is, “when” and “where” a ball is delivered, to succeed in a striking action under high temporal constraints [7]. To understand how expert players can perform such a difficult task under high temporal constraints, previous researchers focused on an expert’s predictive skill based on prior visual information, such as the opponents’ kinematics and ball trajectory using a temporal occlusion paradigm [8–12]. These results demonstrated that expert players could make predictions more quickly and take decisions more accurately using prior visual information compared to novice players [13, 14].

A recent study on an expert’s prediction skill focused on the coupling of visual information and striking action to meet “ecological validity” [15]. These studies revealed that experts could use different types of visual information that are linked to the component phases of the striking motion [13]. Furthermore, experts could perform different coordination patterns for a successful striking action using different visual information [15], suggesting that successful striking is linked to the concept of motor equivalence [16] or redundancy in solutions [17]. The term “redundancy” implies that the motor system generates parallel possible goal solutions [18]. In other words, successful striking has many possible solutions by a variation of shots like a hard or drop shot in striking sports, suggesting that players could perform different striking actions using redundancy in possible solutions even under the same task constraints.

This study deals with a softball game as one of the striking sports. In softball, the player, called a pitcher, who belongs to the defense team, throws a ball. Another player, called a batter, who belongs to the offense team, hits the ball thrown by the pitcher. The batter must hit the ball such that it does not become a determined failure, called “out” as per the rules. Softball is divided into several disciplines depending on how the pitcher throws. We focused herein on “fast-pitch softball,” which involves the highest ball speed, and is adopted in international games [19]. The time duration from the ball thrown by the pitcher to the bat–ball contact was less than 0.5 s. We call this time duration the “ball travel time.” Japanese fast-pitch softball mainly comprises three competition categories: high school, college, and league. These categories are characterized by age and experience of playing softball. Player ages range from 15 to 18 years in the high school category, 18 to 22 years in the college category, and over 18 years in the league category. The experience of playing softball is longer in the order of league, college, and high school. The skill level in the league category would be the highest among the three categories while that of the college category would be higher than that of the high school category. Supporting Information (S1 Fig) shows the details of the first-pitch softball rule.

Softball is characterized by the role of the defensive player (pitcher) and the offensive player (batter) being different. The interpersonal distance between a pitcher and a batter is fixed, unlike in martial arts. Under this task constraint, a pitcher tries to be prevent the batter from successfully hitting the ball; this is done by increasing the ball speed and/or changing the direction of a thrown ball. Meanwhile, a batter must perform a striking motion to the thrown ball in less than 0.5 s of the ball travel time to not fail [20].

Under these task constraints, the preparatory movements are important in succeeding in various striking motions. The preparatory movements are crucial movements for the main movements because they affect the bat–ball contact and outcome. For example, in vertical jump, the preparatory movement (e.g., knee bending) correlates to the jumping height. Similarly, in striking motions, larger preparatory movements are required for a longer ball flight. According to the fast-pitch softball rules in Japan, a rubber ball is used up to high school for safety reasons, while a leather ball is used by college and league players. The ball is created by covering the core part (compressed cork material) with rubber or leather [21]. A leather ball has higher stiffness than a rubber ball. Hence, if a batter hits a rubber ball and a leather ball with the same force, some parts of the energy of the collision with the rubber ball are used for deformation. As a result, the ball flying distance for the leather ball is greater than that for the rubber ball. If a batter aims to obtain the same ball flying distance, a larger preparatory movement is required when hitting a rubber ball compared to hitting a leather ball. The magnitude of the preparatory movement would be affected by these material properties; thus, these properties are considered as tool constraints for the batter.

Previous research showed that the preparatory movement also changes depending on the temporal constraints like ball speed. For example, expert baseball batters exploit earlier visual information such as the throwing movement of the pitcher because the available visual information for the batter decreases corresponding to the decreasing ball viewing time. As a result, the initiation time of expert striking motion is earlier than that of a less expert [22]. These results suggest that experts not only can more accurately predict the ball speed and direction but also perform their striking motion, including the preparatory movement, earlier [13]. Temporal constraints generally correspond to the skill level of the player. Consequently, players who compete at higher competition levels would initiate the striking motion earlier. We define the time duration from the pitcher’s ball release to the bat–ball contact, called “ball travel time,” as a temporal constraint for the batter. According to this definition, the decrease of the ball travel time means an increase in temporal constraints. When the ball travel time decreases, the batter has less time to visually assess the details of the ball, such as the ball trajectory, speed, and location, and successful striking motions would be more difficult. In other words, the temporal constraints for the batter increase in the order of high school, college, and league.

Thus, the initiation time and the magnitude of the preparatory movements would be characterized by the striking motions in softball. As summarized, in terms of the relationship between the task constraints and the preparatory movements for batters in softball, greater temporal constraints affect the initiation time of the preparatory movements, and high tool constraints (e.g., material properties of the ball) affect the magnitude of the preparatory movements.

We examined the effects of temporal and tool constraints on the preparatory movement by investigating the striking motion of fast-pitch softball to understand how players solve the task as a task-oriented behavior under various task constrains. To this end, we compared the preparatory movements of Japanese softball players among different competitive categories, which also have different temporal and tool constraints.

**Fig 1:**
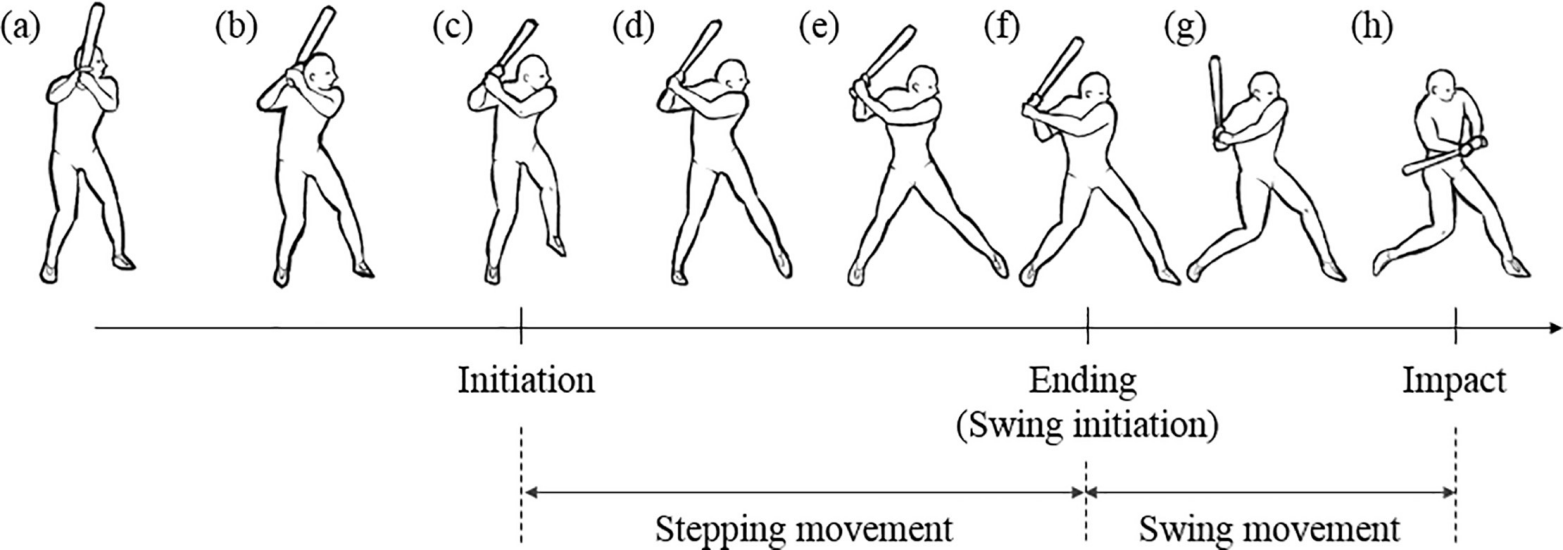
**Series showing a batter’s striking motion**: (a)–(b) observation of the pitcher’s motion; (c) initiation of the preparatory movement (Initiation); (d)–(e) weight shift to forward and taking backswing; (f) ending of the preparatory movement (Ending) and swing initiation; (g) forward swing; and (h) bat–ball contact (Impact).

Fig 1 shows a series of a batter’s striking motion. We define the two legs of the batter as the front leg and the hind leg. The batter’s front leg is closer to the pitcher than the batter’s hind leg. The batter observes the pitcher’s movement before the striking motion (Fig 1A and 1B). The striking motion can be divided into two movements: stepping movement (Fig 1C–1F) and swing movement (Fig 1F–1H) corresponding to the preparatory and main movements, respectively. The preparatory movement can be defined from the raising front leg to the landing front leg, called “initiation” (Fig 1C) and “ending” of the stepping movement (Fig 1F), respectively. The main movement can be defined to start from the ending of the stepping movement (Fig 1F) to the bat–ball contact, called “impact” (Fig 1H). The initiation of the stepping movement defined as the “initiation time” is the initiation time of the preparatory movement. The duration of the stepping movement defined as the “step duration” is the magnitude of the preparatory movement. These two variables are the dependent variables used herein to investigate the stepping movement of fast-pitch softball hitting.

**Fig 2:**
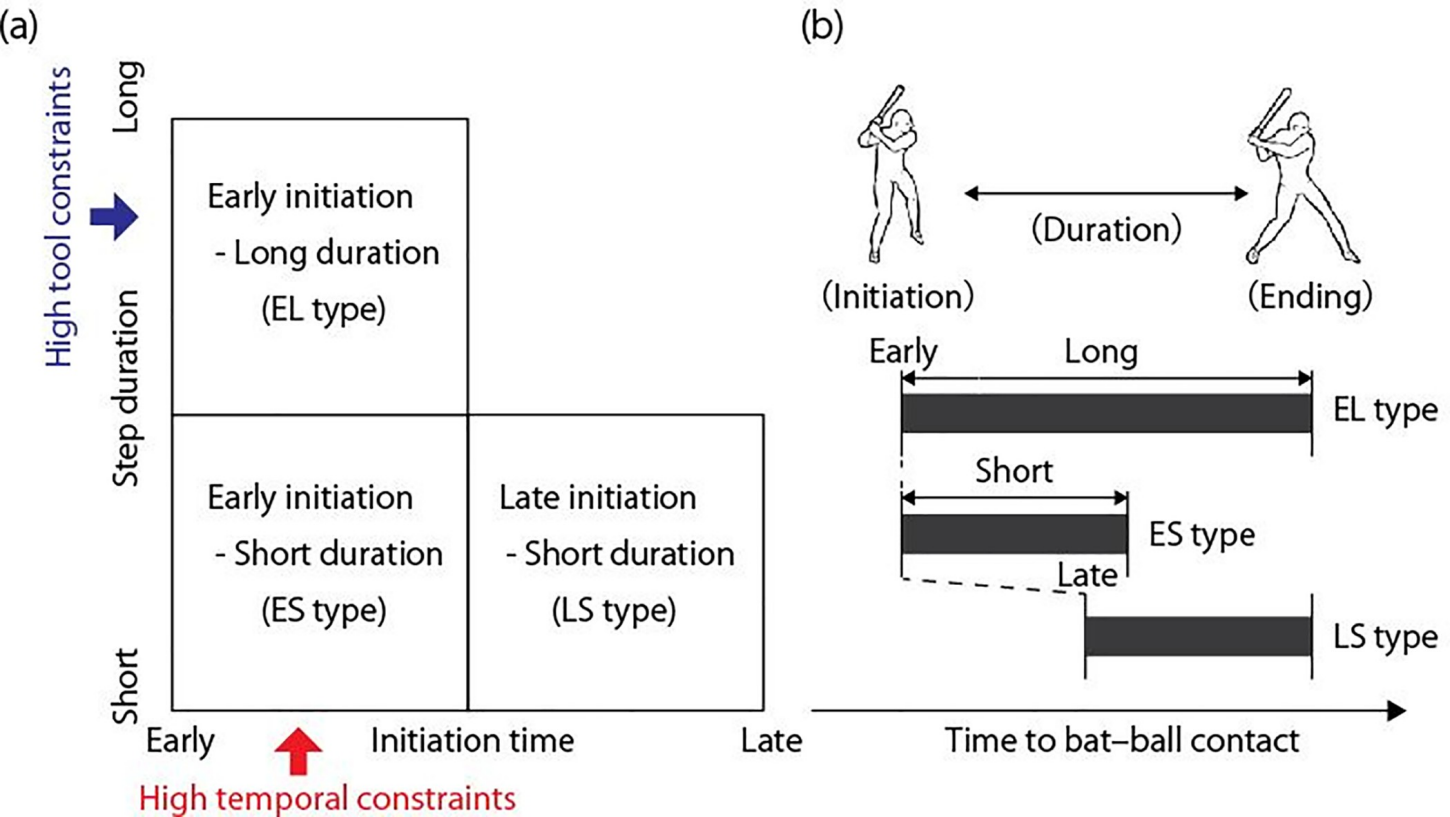
(a) Three types of stepping movement in the initiation time–step duration plane and (b) schematic of the three types of stepping movement.

The stepping movement pattern could be characterized by the function of the “initiation time” of the preparatory movement and “step duration” as the magnitude of the preparatory movement. Thus, we can classify the stepping movement pattern for each batter using the “initiation time” and the “step duration” corresponding to the temporal and tool constraints, respectively. High temporal constraints would cause an early initiation of the stepping movement, whereas high tool constraints would lengthen the step duration of the stepping movement. As shown in Fig 2, the stepping movement performed by the batter could be divided into three types based on the combination between the initiation time and the step duration variables and the relationship between the task constraints and the step movement: late initiation–short duration (LS), early initiation–long duration (EL), and early initiation–short duration (ES) types. Possible combinations include late initiation–long duration (LL type); however, performing a long step duration from a later initiation timing implies that a sufficient time for swing movement cannot be secured (Fig 1).

Taken together, we could hypothesize the pattern of the preparatory movements in each competition category based on the relationship between the task constraints in each category and their preparatory movements. The high school category would have low temporal constraints and high tool constraints. High tool constraints would cause a long step duration of the stepping movements, whereas low temporal constraints would not affect the initiation time of the stepping movement. As a result, the players in this category would demonstrate a long duration of the stepping movement corresponding to the high tool constraints and perform both early and late initiations of the stepping movements. However, as mentioned earlier, the batter who belongs to the LL type would find it difficult to successfully strike the ball, resulting in a high proportion of the EL type. The college category would have intermediate temporal constraints and low tool constraints. Low tool constraints would not affect the step durations of the stepping movement, while intermediate temporal constraints could cause both early and late initiations of the stepping movements. As a result, the players in this category would show all three types of stepping movements. The league category would have high temporal constraints and low tool constraints. High temporal constraints would cause an early initiation of the stepping movement, whereas low tool constraints would not affect the step duration of the stepping movements. The players in this category would show early initiation and short duration of the stepping movement (ES type) or early initiation and long step duration (EL type) corresponding to high temporal constraints.

Overall, from the abovementioned theoretical background, our purpose is to clarify the relationship between the preparatory movement and the task constraints in each competition category and obtain more insights about the motor solution under task constraints. We particularly examine the following five hypotheses:

1. The temporal constraints will increase in the order of high school, college, and league.
2. The initiation time will decrease corresponding to the high temporal constraints.
3. The step duration will increase corresponding to the high tool constraints.
4. The stepping movement will be classified into three types (i.e., EL, ES, and LS) based on the combination of the initiation time and the step duration in fast-pitch softball hitting corresponding to the temporal and tool constraints.
5. The players in each category would be classified into a particular type of the stepping movement corresponding to the temporal and tool constraints in each category.

## Method

### Participants

The participants in the present study were high school softball players, who participated in the 2017 Japanese High School Softball Invitational Tournament (March 19–20, 2017), collegiate players, who participated in the 2016 Spring College Women’s Softball League in the Tokai district (April 30, May 1, 3, 2016), Japan, and league players, who participated in the 2016 Japan Women’s Softball League (May 14–15, 28–29, 2016). These teams are top-level teams in each competition category, and we believe that they are well adapted to each task constraint.

The participants specifically comprised players from 13 high school teams, nine college teams, and nine league teams in Japan. The number of participants was 121 high school players (105 batters and 16 pitchers), 114 college players (95 batters and 19 pitchers), and 120 league players (98 batters and 22 pitchers). All participants were female. Unfortunately, we could not obtain detailed data on the age and years of experience of the participants, but we could assume that high school students were aged 15 to 18 years; college students were 18 to 22 years; and league players were 18 to 35 years (35 years is the highest age among players in the league). The years of experience were at least 3 to 6 years for high school students, 6 to 10 years for college students, and 6 to 23 years for league players.

### Recording setup

**Fig 3:**
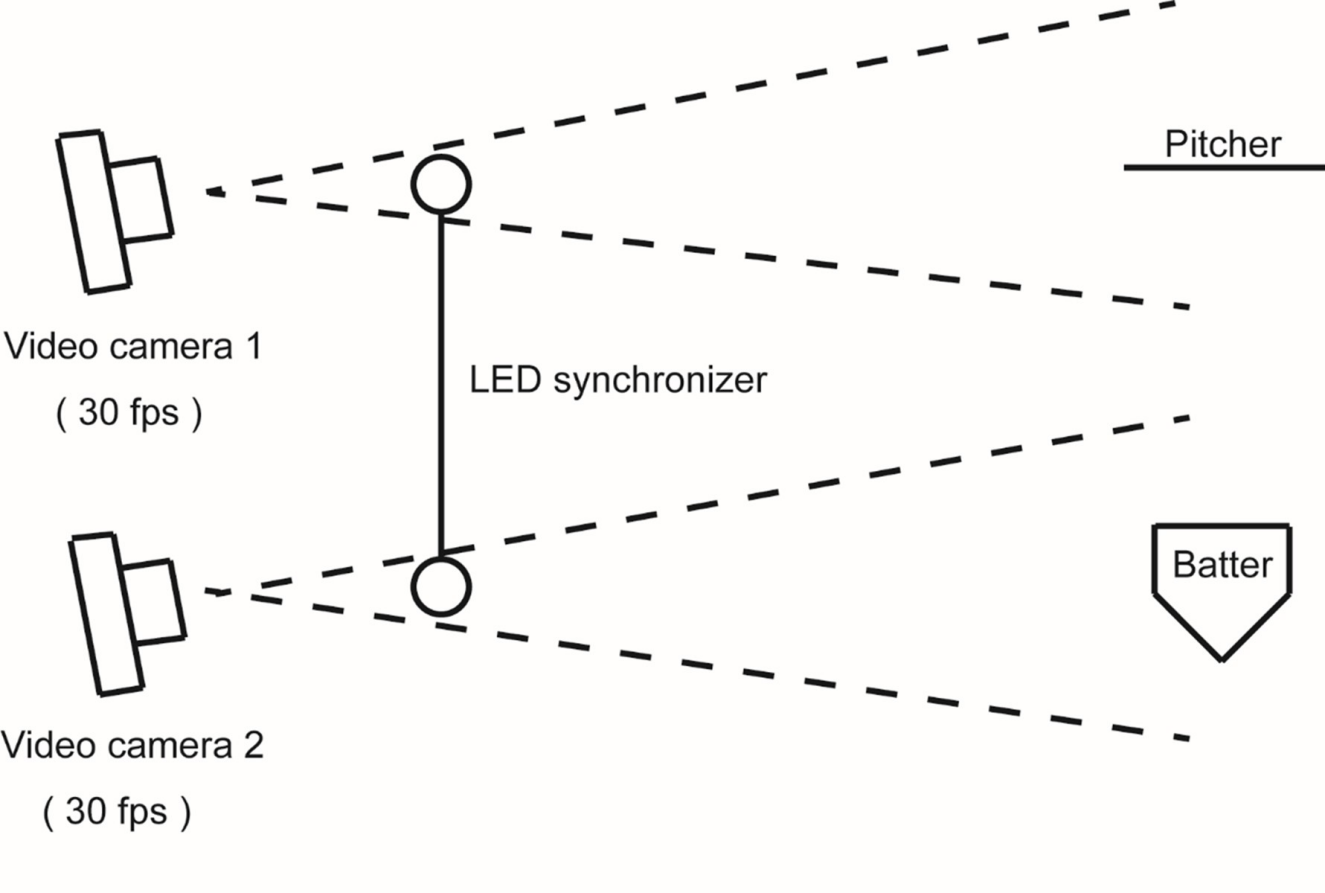
Schematic of the video recording setup.

Fig 3 shows the setup used for the video recording. The throwing and striking motions of a game were recorded by two 30-fps video cameras (Sony HDR-XR550, Sony HDR-CX360V). An LED synchronizer (PTS-110, DKH Co., Ltd., Japan) was used to synchronize two simultaneous video recordings by turning a light on/off within two visual fields. Both cameras were positioned at the third- or first-base side stand, with one camera recording the throwing movement and the other recording the hitting movement.

### Procedure

One or two official games of each team were recorded on video. Supporting Information (S1, S2 and S3 Tables) shows the information on the recorded games. For teams playing multiple games according to the round robin or tournament schedule, we analyzed the game with the starting pitchers, who threw the most balls in each team. We analyzed batters, who performed the stepping movement to the thrown ball for more than nine balls in one game, not including bunt, which is intentionally tapping the ball without any stepping movement, and slap hitting, which is hitting while running. Overall, the movements of 122 batters (i.e., 41 high school, 36 college, and 45 league players) were analyzed.

We also measured the ball travel time for pitchers, who played more than four innings among the starting pitchers. The ball travel times of 32 pitchers (i.e., 13 high school, nine college, and 10 league players) were analyzed.

The procedures were approved by the Internal Review Board of the Research Center of Health, Physical Fitness, and Sport at Nagoya University and conformed with the principles of the Declaration of Helsinki. All study participants provided informed written consent.

### Measurements

#### Determining the movement event

**Fig 4:**
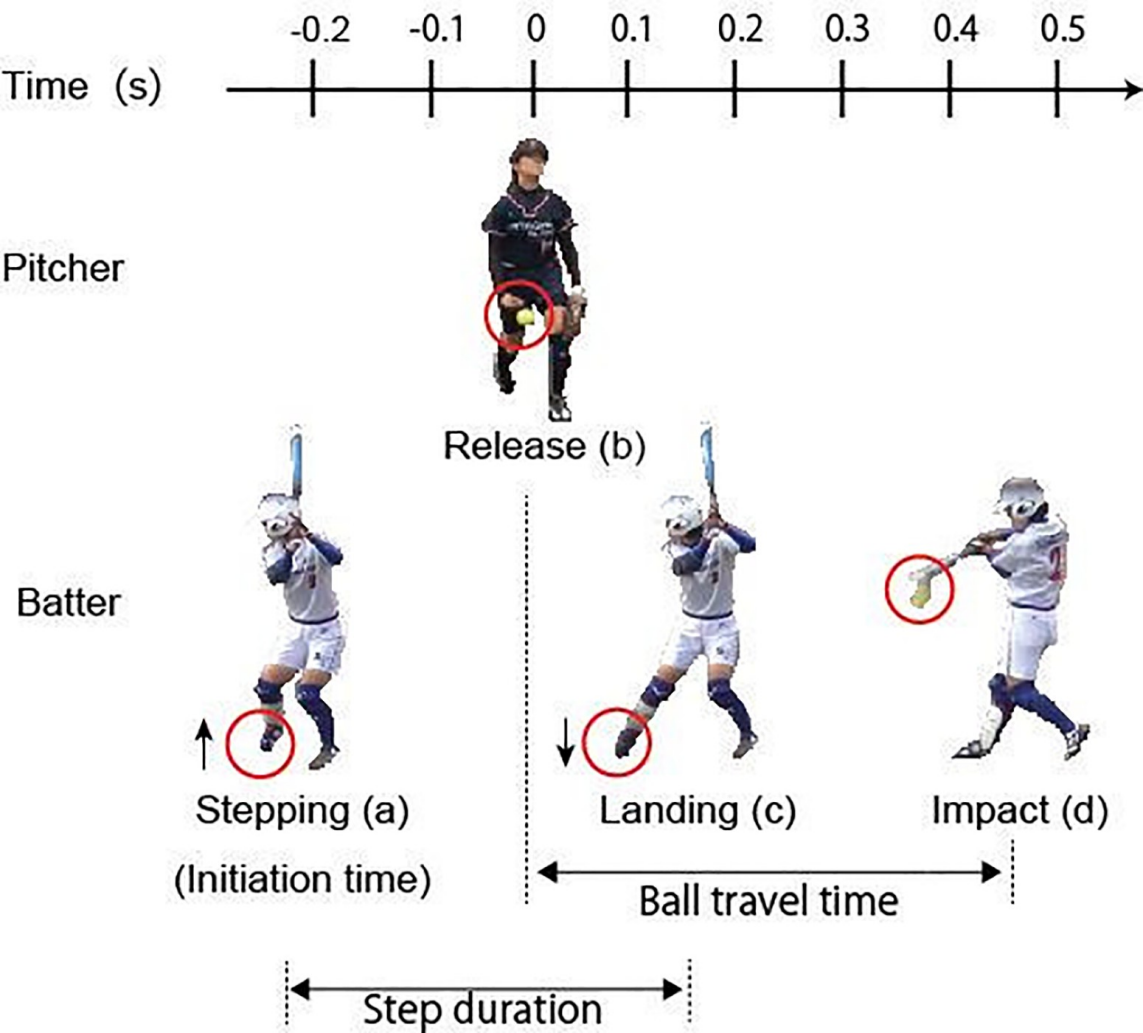
**Four identified events**: (a) initiation, (b) release, (c) ending, and (d) impact. (a), (c), and (d) are movements of the batter, whereas (b) is the movement of the pitcher.

The video editing software EDIUS (EDIUS Neo version 2.5, Grass Valley Co. Ltd.) was used to load the video data, separate the frame-by-frame images, and synchronize the two video recordings based on the LED light information. The frame-by-frame images were used to determine the timing of four events, namely initiation, ending, release, and impact at 30 fps (Fig 4). The occurrence time of the initiation was identified when the batter lifts her front leg from the ground while that of the ending event was identified when she places her front leg on the ground after initiation. The occurrence time of each event was expressed with reference to the release of the ball by the pitcher to clearly show how batters perform the stepping movement against the pitcher. In cases when two or three stepping movements were observed during one throwing of the pitcher, the last stepping movement was selected for analysis. The release time was identified when the ball was released from the pitcher’s hand, whereas the impact time was identified when the ball collided with the bat and changed direction. A 30-fps measurement would be insufficient in identifying the ball release and impact; hence, the ball release by the pitcher and the impact of the batter were measured at 60 fps by deinterlacing the videos. We were sometimes unable to identify the precise times of the release and/or impact events. In such cases, the release time was identified as one frame before the ball was first observed to have been released from the pitcher’s hand while the impact time was similarly identified as one frame before the ball was observed to have changed direction.

#### Ball travel time as temporal constraints

We defined the time duration from the pitcher’s ball release (release event) to the bat–ball contact (impact event), called ball travel time, as the temporal constraints for the batter to confirm the relationship between the competition categories and the temporal constraints (Fig 4). We calculated the average ball travel time of the starting pitcher for each game to examine the temporal constraints of each competition category.

#### Dependent variables of the stepping movement

We defined two dependent variables to examine the characteristics of the stepping movement (Fig 4). The first variable is the initiation time, which is the moment at which the stepping movement of the opposing pitcher begins and is the same as the initiation time of the initiation event against the pitcher’s ball release. The second variable is the step duration, which is the duration of the stepping movement that can be calculated by subtracting the occurrence time of the ending event from that of the initiation event. The release time was set to 0; hence, a negative value of the initiation time indicates that a batter initiated her stepping movement before the ball was released by a pitcher. These two variables were calculated for each batter and each ball thrown by the pitcher. The average value for nine hitting actions of each batter was adopted as the representative value for the batter. A local outlier factor method [23] was applied to two variables (i.e., initiation time and step duration) of the three competition categories to eliminate the effects of the outliers.

## Data analysis

The data for two high school batters were eliminated based on the results of the application of the local outlier factor method. A total of 120 batters (39 high school, 36 college, and 45 league batters) were analyzed overall.

We used statistical analysis to verify our hypothesis, wherein we first verified Hypothesis 1 using one-way analysis of variance (ANOVA) to examine the differences in the ball travel time of the three competition categories. Post-hoc (Bonferroni) pairwise comparisons were used to determine the statistically significant differences between each competition category. The significance level was set at 0.05. The effect size *d* (Cohen's *d*) was also calculated.

Next, we verified hypotheses 2 and 3 by performing one-way ANOVA to examine the differences between the initiation time and step duration of the three competition categories. Post-hoc (Bonferroni) pairwise comparisons were used to determine the statistically significant differences between each competition category. The effect size *d* (Cohen's *d*) was also calculated.

We clarified Hypothesis 4 through hierarchical cluster analyses of the two dependent variables to classify the stepping movement patterns of each batter. In the cluster analysis of a variable, the high school, college, and league players were pooled for classification by the common criteria among the three categories. Hierarchical cluster analysis is widely used for data mining to determine the hierarchic cluster of the given data. The results are usually presented in a dendrogram. Ward’s minimum variance method [24] and Euclidean distance were employed herein. Ward’s minimum variance method generates the clusters in a manner that minimizes the intra-cluster variance. It is favored for its high classification ability [25, 26].

The primary drawback of hierarchical cluster analysis is determining the number of clusters to use in presenting the results. The present study used the Jain–Dubes method to determine the optimal number of clusters [27]. The method identifies the optimal number of clusters, *k*, that minimizes the evaluation function p(*k*), which can be expressed as follows:

$$p(k)=\frac{1}{k}\sum_{i=1}^{k}\underset{i\leq j\leq k}{\max}\left\{\frac{\eta_{i}+\eta_{j}}{\xi_{ij}}\right\}$$ (1)

where *k* is the number of clusters (2≤*k*≤log_2_ *n*, *n* is the number of data points); *η*_*i*_ is the average distance between the data points in cluster *i*; and *ξ*_ij_ is the distance between clusters *i* and *j*. The cluster analysis classified the stepping movement of all the batters into several patterns.

Finally, we clarified Hypothesis 5 by performing a Fisher’s exact test to examine the relationship between the task constraints of each competition category and the stepping movement pattern. If Fisher’s exact test reveals a significant difference, a pairwise Fisher’s exact test is conducted to determine which comparisons are statistically significant in each competition category and stepping pattern. The Bonferroni adjustments of the *p*-value were employed for the pairwise Fisher’s exact test.

### Results and discussion

#### Temporal constraints in each competition category (Hypothesis 1)

**Fig 5:**
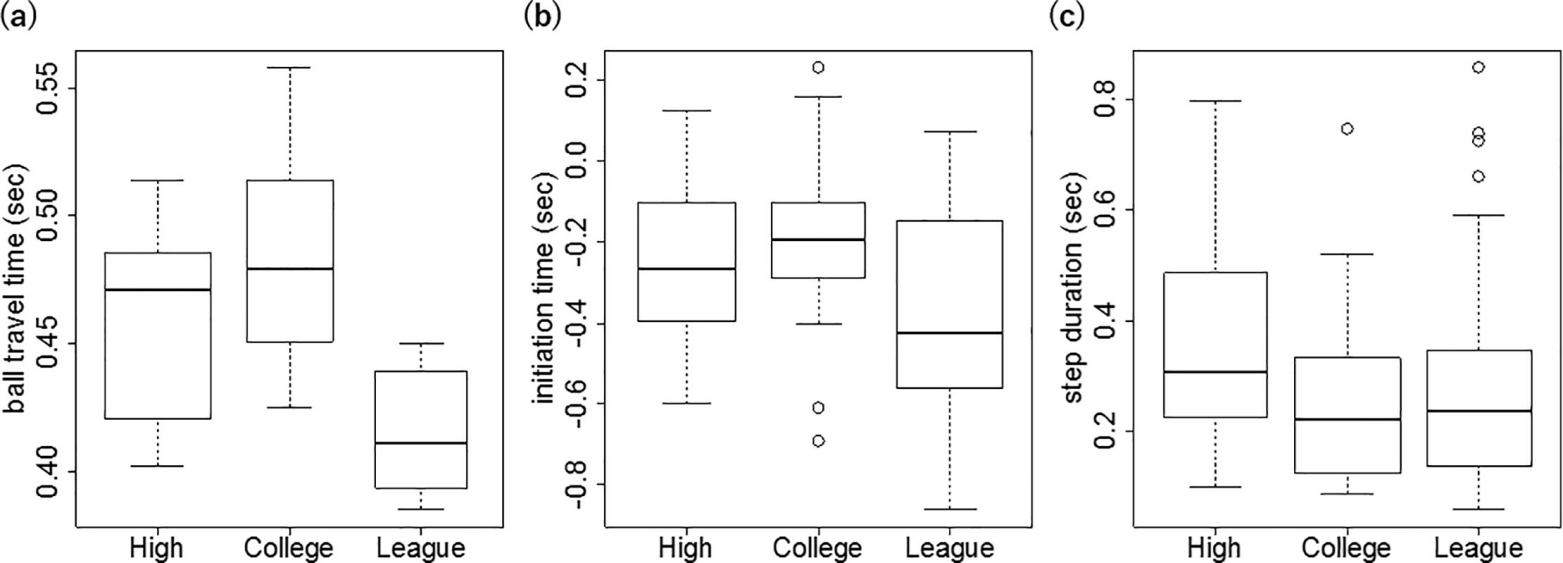
**Boxplots of the ball travel time (a), initiation time (b), and step duration (c) for the three competition categories**.

Fig 5(A) shows the boxplots of the ball travel time for each competition category. The average ball travel time for the high school players was 0.46 ± 0.04 s (*n* = 13), while those for college and league players were 0.48 ± 0.05 s (*n* = 9) and 0.41 ± 0.02 s (*n* = 9), respectively. One-way ANOVA revealed significant differences among the ball travel times of the three competition categories (*F*(2, 29) = 3.32, *p* = 1.9 × 10^−3^). The post-hoc Bonferroni pairwise comparisons revealed that league players had a significantly shorter ball travel time than did high school (t = 3.60, *p* = 1.6 × 10^−3^, *d* = 1.45) and college (t = 3.62, *p* = 3.4 × 10^−3^, *d* = 1.80) players, whereas no significant difference was found between high school and college players (t = 0.94, *p* = 0.35). Therefore, the temporal constraints of the league pitcher were the highest. In contrast, no difference was found in the ball travel time between high school and college players, suggesting that Hypothesis 1 was partially supported. The temporal constraints in the league category were the highest compared to the college and high school categories. However, contrary to our hypothesis, the temporal constraints in college and high school were the same. The average ball travel times for all the pitchers is given in the Supporting Information (S4, S5 and S6 Tables).

The determined average ball travel time of 0.41 s for the league players corresponded to a ball speed of more than 95 mph in baseball [20]. Compared to cases where the experiments were performed in a laboratory, where the time duration was approximately 0.50 s or longer [28, 29], the present result indicated severe temporal constraints. These results showed that the striking motion of the league players herein was under different temporal constraints compared to those in conventional experimental settings.

However, these results were of the 60-fps measurements; thus, we believe that caution is needed in the interpretation. The study results showed no significant difference in the temporal constraints between high school and college players. However, a difference smaller than the temporal resolution used in this research may be observed.

#### Temporal constraints and initiation time (Hypothesis 2)

Fig 5(B) shows the boxplots of the initiation time for each competition category. The average initiation time for the high school players was −0.26 ± 0.18 s (*n* = 39), while the corresponding values for the college and league players were −0.19 ± 0.18 s (*n* = 41) and −0.38 ± 0.25 s (*n* = 45), respectively. One-way ANOVA revealed significant differences among the initiation times of the stepping movements in the three competition categories (F(2, 117) = 8.42, *p* = 3.84 × 10^−4^). A post-hoc Bonferroni pairwise comparison also revealed that the initiation time of the league players was significantly earlier than those of the high school (t = 2.34, *p* = 0.032, *d* = 0.51) and college (t = 3.81, *p* = 4.0 × 10^−4^, *d* = 0.85) players, whereas no significant difference was observed between high school and college (t = 1.82, *p* = 0.11) players, suggesting that Hypothesis 2 was supported. These results showed that the initiation time of the stepping movements of the league players with higher temporal constraints was earlier than those of the two other categories. Moreover, no significant differences were found between the high school and college categories because the temporal constraints in these two categories were equal. Thus, the initiation times were earlier corresponding to the high temporal constraints.

As mentioned in the Introduction section, previous studies on the striking motion similarly reported that expert players with high temporal constraints begin their preparatory movement earlier than less expert ones [22]. For a higher ball speed, the batter has less available visual information to assess the information about the ball (e.g., ball trajectory). Thus, expert batters exploit earlier visual information and integrate it with their leg movement [13] and tend to initiate their movement earlier. The results of this study revealed that the early initiation of the preparatory movement under high temporal constraints can be observed not only under experimental settings, but also in real-life situations.

#### Tool constraints and step duration (Hypothesis 3)

Fig 5(C) shows the boxplots of the step duration of the stepping movements for each competition category. The average step duration for the high school players was 0.38 ± 0.20 s (*n* = 39), while the corresponding values for the college and league players were 0.22 ± 0.11 s (*n* = 41) and 0.29 ± 0.20 s (*n* = 45), respectively. One-way ANOVA revealed significant differences among the step duration of the three competition categories (F(2, 117) = 7.69, *p* = 7.27 × 10^−4^). The post-hoc Bonferroni pairwise comparison also revealed that high school players had a significantly longer step duration than college players (t = 4.25, *p* = 9.16 × 10^−5^, *d* = 0.98) and a marginally longer step duration than league players (t = 2.08, *p* = 0.059, *d* = 0.46). No significant difference was found between college and league players (t = 1.85, *p* = 0.10), suggesting that Hypothesis 3 was partially supported. The high school category used a rubber ball, while the college and league categories used a leather ball. Thus, the tool constraints in the high school category were higher than those in the other two categories. The results showed that the step duration in the high school players was longer than those in the college and league players, corresponding to the tool constraints.

The relationship between the required impact velocity and the preparatory movement in the striking motion was investigated experimentally. Caljouw et al. [30, 31] compared the striking motions targeting near and distant goals. The results showed that when participants targeted distant goals, the hitting tool was accelerated at the time of impact, and the participants needed more preparation time, suggesting that high school players need longer preparatory durations to cope with high tool constraints and obtain a long ball flying distance.

#### Stepping movement pattern of fast-pitch softball (Hypothesis 4)

**Fig 6:**
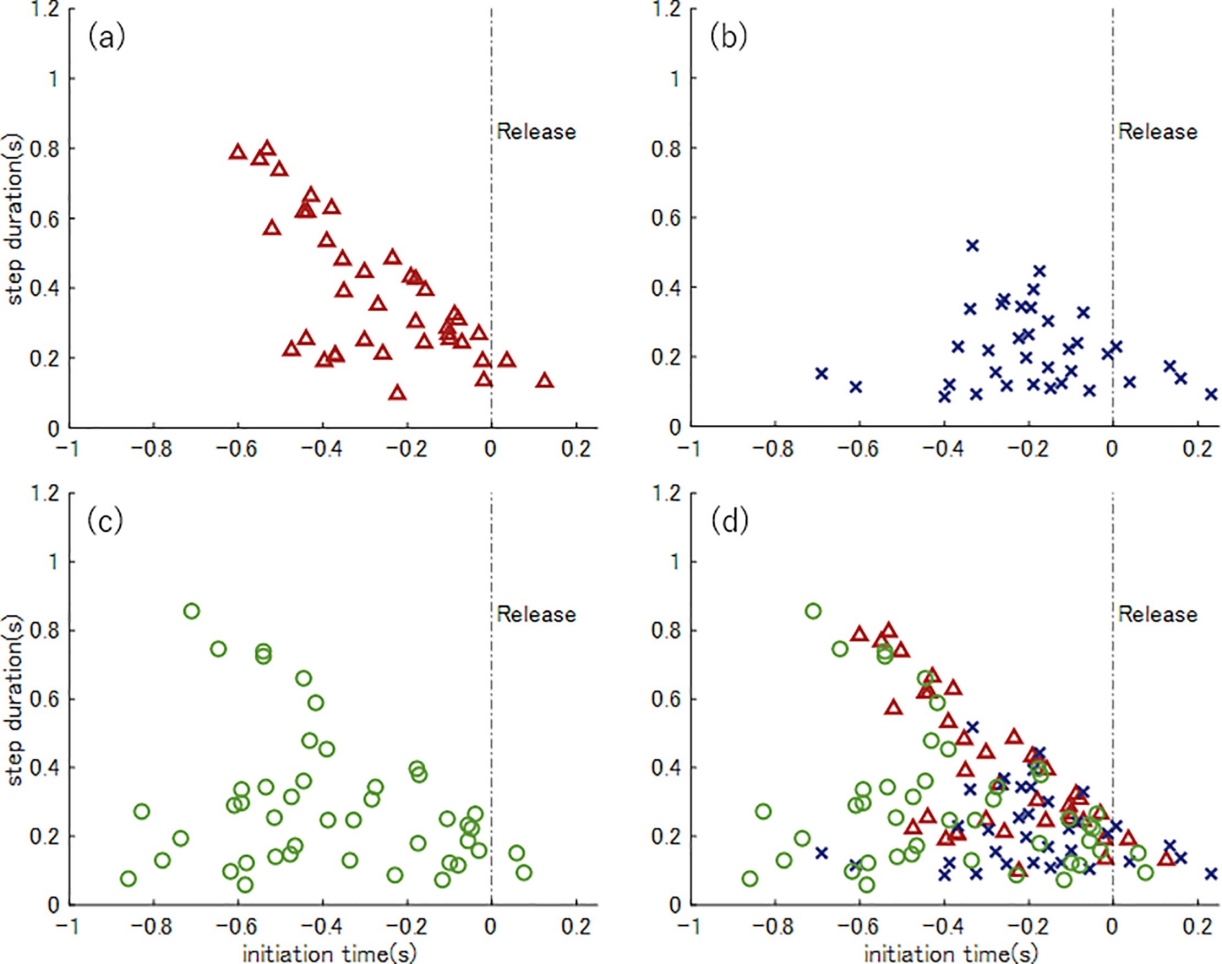
Relationships between the initiation time and the step duration for high school (a), college (b), and league players (c). Comparison of all the data for all the three player categories (d). Triangles, crosses, and circles represent the high school, college, and league players, respectively.

**Fig 7:**
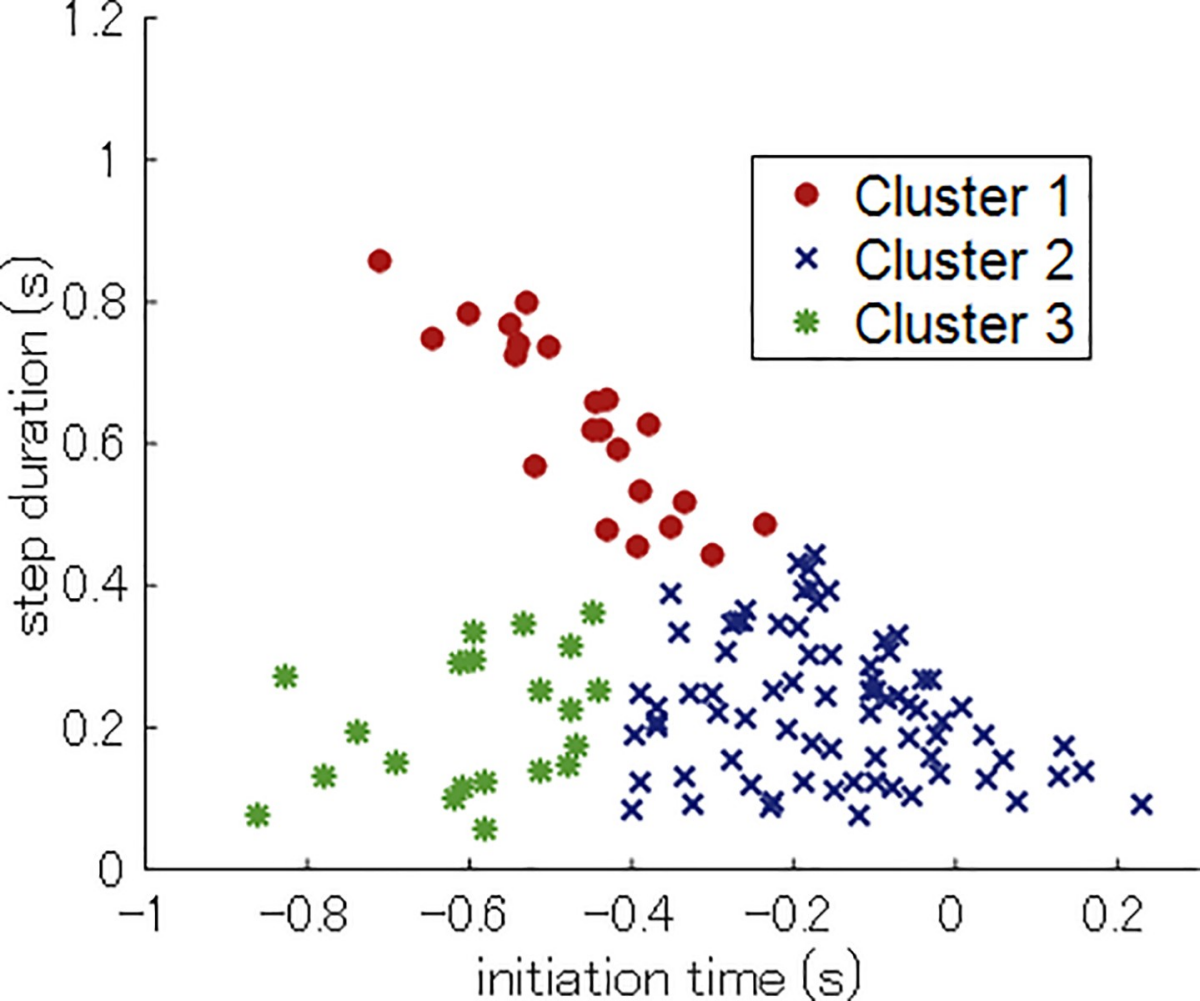
Three clusters of the stepping movement.

Fig 6(A)–6(C) show the relationships between the initiation time and the step duration for the high school, college, and league players. Individual players for the three competition categories are superposed in Fig 6(D), with each data point representing the stepping movement patterns for the individual players. A data point (−0.2, 0.4), for example, indicated that the batter initiated the stepping movement 0.2 s before the ball release by the pitcher and landed their front leg 0.4 s after the initiation (0.2 s after ball release). The optimal number of clusters, *k*, for the present data was determined as three (evaluation function *p*(3) = 0.66) using the Jain–Dubes method. We show the detailed process for determining the number of clusters in Supporting Information (S2 Fig). Fig 7 depicts the three clusters obtained in the stepping movements of all the players.

The results obtained suggest that Hypothesis 4 was supported. As per our original hypothesis, the cluster analysis revealed three optimal clusters in the stepping movement of softball players corresponding to temporal and tool constraints. In other words, cluster 1 corresponded to the EL type; cluster 2 corresponded to the LS type; and cluster 3 corresponded to the ES type.

#### Task constraints of each competition category and stepping pattern (Hypothesis 5)

**Fig 8:**
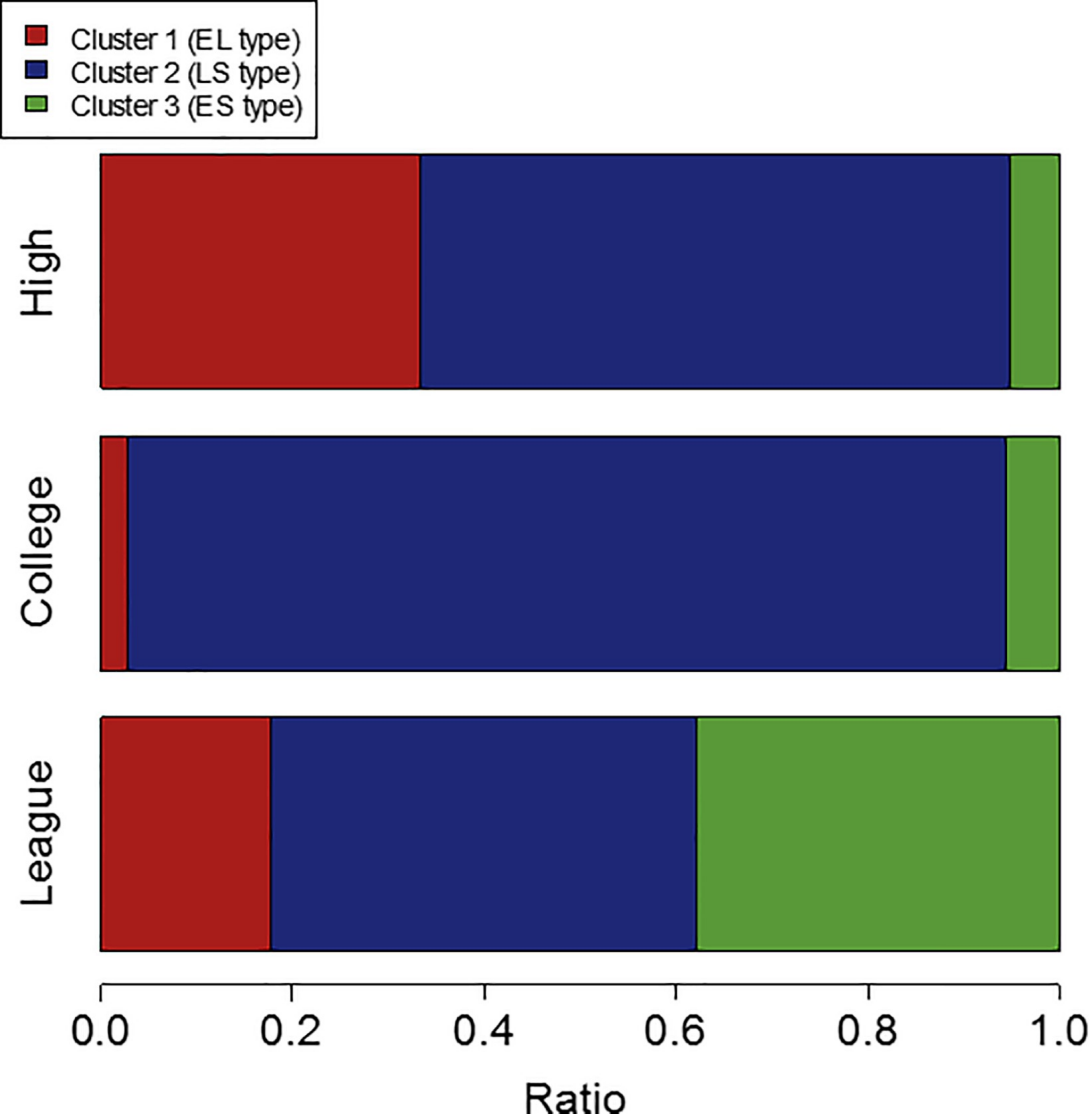
Ratios of the players in different clusters in each competition category.

Table 1 lists the number of players in each cluster for each category, while Fig 8 illustrates a bar chart of the ratios of the players in the different clusters in each competition category. From the 3 (cluster) × 3 (competition category) contingency table, significant differences were observed among the frequencies of the stepping patterns classified by the cluster analyses of each competition category (*p* = 6.76 × 10^−7^). A pairwise Fisher’s exact test revealed that the frequency of the stepping movement pattern of the high school players was significantly higher in cluster 1 (EL type) and significantly lower in cluster 2 (LS type) compared to that of the college players (*p* = 2.03 × 10^−3^), while that of the college players was significantly higher in cluster 2 (LS type) and significantly lower in clusters 1 (EL type) and 3 (LS type) compared to that of the league players (*p* = 0.02 and 3.38 × 10^−4^, respectively). That of the high school players was significantly higher in clusters 1 (EL type) and 2 (LS type) and significantly lower in cluster 3 (ES type) compared to that of the league players (*p* = 1.06 × 10^−3^ and 1.69 × 10^−3^, respectively).

**Table 1:** Frequency of the stepping pattern of each competition category in each cluster.

	Cluster 1 EL type		Cluster 2 LS type		Cluster 3 ES type	
Category	Number	Ratio	Number	Ratio	Number	Ratio
High school	13	0.33	24	0.62	2	0.05
College	1	0.03	33	0.92	2	0.06
League	8	0.18	20	0.44	17	0.38

The numbers of major types of stepping movements shown in the high school, college, and league categories were two (EL and LS), one (LS), and three (all types), respectively (Fig 8), suggesting that Hypothesis 5 was partially supported. These results showed that the preparatory movement differed for each competition category even in the same sports and was affected by the task constraints in each category.

In our hypothesis, we assumed that the high school category would have low temporal constraints and high tool constraints. Consequently, high school players would have a high ratio of the EL type. The results partially supported this hypothesis on the high school category. However, high school players also performed the LS type of the stepping movement. Contrary to our hypothesis on the college category, college players with low temporal and tool constraints showed only one type of stepping movement; that is, the LS type. Finally, as in our hypothesis, the league category had a high proportion of the ES or EL type. However, some players showed an LS type with late initiation time despite the high temporal constraints. Overall, as in our hypothesis, high temporal constraints resulted in high proportions of the EL and ES types, and high tool constraints resulted in high proportion of the EL type. However, the LS type was observed in every category despite the high temporal or tool constraints. The LS type was observed in every category because of the redundancy of the successful striking motion.

The inter-individual differences in the movement initiation of the expert players have been reported in several studies [16]. The advantage of the LS type is the long viewing time for a pitcher’s kinematics and the thrown ball by a pitcher. A batter would want to obtain more information on the thrown ball’s trajectory, speed, and direction before beginning their preparatory movement. The late initiation of the stepping movement can enable a longer viewing time for a batter; however, the short duration of the stepping movement cannot be performed to produce a larger force at the bat–ball contact. However, batters in softball do not necessarily need to produce a larger force every time. In other words, they have to avoid a determined failure “out” in the rule; that is, unsuccessful hitting. Under these task constraints in softball, players in every category could adopt the not-to-lose strategy using the redundancy in solutions. Particularly in college players, many players in every category tended to choose the LS-type stepping movement because they do not necessarily need to perform early initiation and produce a larger force under task constraints (e.g., task-oriented behavior).

## General discussion

This study aimed at clarifying the relationship between the task constraints of different competition categories and the stepping movement of fast-pitch softball batters in Japan. Table 2 summarizes these results. First, we investigated how temporal constraints differ in each competition category (Hypothesis 1). The results demonstrated significant differences between the league and the other two categories. These temporal and tool constraints affected their stepping movements in terms of both initiation time and step duration (hypotheses 2 and 3). The cluster analysis using two dependent variables comprising the initiation time and the step duration revealed three types of stepping movement, that is, LS, EL, and ES types in fast-pitch softball (Hypothesis 4). The players in each category were classified into a particular type of stepping movement corresponding to the temporal and tool constraints in each category (Hypothesis 5). However, high school players performed using the EL and LS types; college players were classified into only the LS type; and league players were classified into all three types of stepping movement.

**Table 2:** Relationship between the task constraints and the stepping movement of fast-pitch softball hitting for each category.

Competition category	Temporal constraints (ball travel time)	Tool constraints (ball material)	Types of stepping movement
High school	Low (longer)	High (rubber ball)	EL and LS
College	Low (longer)	Low (leather ball)	LS
League	High (shorter)	Low (leather ball)	EL, ES, and LS

Contrary to our hypothesis, many players in every category demonstrated late initiation and short duration of the stepping movement even under higher temporal constraints in the league category. The late initiation of the stepping movements enabled the player to obtain more information on the ball trajectory, speed, and direction to anticipate “when” and “where” a ball is delivered more accurately before their preparatory movement begins. As a result, they perform late initiation to strike a ball successfully. In addition, batters would avoid unsuccessful hitting; thus, producing a larger force every time is unnecessary. They would perform their striking motions accurately with a short duration of the preparatory movement. In contrast, when batters initiate their stepping movement early, they could have a long duration of the stepping movement to produce a larger force while exploiting earlier visual information. In other words, batters in softball show individual differences for successful hitting; that is, they exploit a redundancy in solutions under various task constraints. They choose their preparatory movements as a not-to-lose strategy under various task constraints instead of risk taking [1]. This suggests that players in every category solve their tasks by exploiting the redundancy in solutions in the task space as a task-oriented behavior [32]. We consider that these results apply not only to fast-pitch softball, but also to other sports.

However, we cannot deny the possibility that factors, which we did not focus on in this study, such as age and experience playing softball, affect these results. Despite the fact that age and experience playing softball increased in the order of high school, college, and league, the number of stepping movement patterns increased in the order of college, high school, and league. This research is only a cross-sectional study of the relationship between the task constraints and the stepping movement for the high school, college, and league levels. Hence, we need a longitudinal study to clarify the detailed process from which these differences are obtained.

## Conclusion

This study aimed to clarify the relationship between the task constraints and the preparatory movement of fast-pitch softball batters in the three Japanese competition categories. The players in each category with their respective temporal and tool constraints showed a particular type of stepping movement as a preparatory movement corresponding to each task constraint. However, some players in every category showed late initiation and short step duration of the stepping movements even if under high temporal constraints. They chose their preparatory movements as a not-to-lose strategy under various task constraints, which suggests that they solve tasks by exploiting the redundancy in solutions in the task space under task constraints.

## Supporting information

S1 Fig Softball field. (TIFF)

S2 Fig p(k) value with respect to cluster number as determined by the Jain–Dubes method. (TIF)

S1 Table List of measured high school games. The “Measure” column indicates the team analyzed in this study (both: both teams 1 and 2 are analyzed; H: only one team is analyzed).
(PDF)

S2 Table List of measured college league games. The “Measure” column indicates the team analyzed in this study (both: both teams 1 and 2 are analyzed; C1, C2: only one team is analyzed).
(PDF)

S3 Table List of measured league games. The “Measure” column represents the team analyzed in this study (both: both teams 1 and 2 are analyzed; L: only one team is analyzed).
(PDF)

S4 Table Number of pitches and average ball travel time of high school pitchers. (PDF)

S5 Table Number of pitches and average ball travel time of college league pitchers. (PDF)

S6 Table Number of pitches and average ball travel time of league pitchers. (PDF)

S1 Supporting Information (DOCX)

## References

[1] Kijima A., Kadota K., Yokoyama K., Okumura M., Suzuki H., and Yamamoto Y. Switching dynamics in an interpersonal competition brings about ‘‘deadlock” synchronization of players. PLoS One. 2012;7: e47911 10.1371/journal.pone.0047911 23144834 PMC3489899

[2] Okumura M., Kijima A., Kadota K., Yokoyama K., Suzuki H., and Yamamoto YA critical interpersonal distance switches between two coordination modes in kendo matches. PLoS One. 2012;7: e51877 10.1371/journal.pone.0051877 23284799 PMC3527480

[3] Okumura M., Kijima A., and Yamamoto Y. Perception of affordances for striking regulates interpersonal distance maneuvers of intermediate and expert players in kendo matches. Ecol Psychol. 2017;29: 1–22.

[4] Yamamoto Y., Yokohama K., Okumura M., Kijima A., Kadota K., and Gohara K. Joint Action Syntax in Japanese Martial Arts. PLoS One. 2013;8: e72436 10.1371/journal.pone.0072436 24023740 PMC3762806

[5] Yokoyama K., and Yamamoto Y. Three people can synchronize as coupled oscillators during sports activities. **PLoS** Comput Biol. 2011;7: e1002181 10.1371/journal.pcbi.1002181 21998570 PMC3188505

[6] Yokoyama K., Shima H., Fujii K., Tabuchi N., and Yamamoto Y. Social forces for team coordination in ball possession game. Physical Review E. 2018;97: 022410 10.1103/PhysRevE.97.022410 29548247

[7] Brenner E. and Smeets JBJ. Hitting moving targets: Co-operative control of “when” and “where”. Hum Mov Sci. 1996;15: 39–53.

[8] Abernethy B. Anticipation in squash: Differences in advance cue utilization between expert and novice players. J Sports Sci. 1990;8: 17–34. 10.1080/02640419008732128 2359149

[9] Abernethy B., Gill D. P., Parks SL., and Packer ST. Expertise and the perception of kinematic and situational probability information. Perception, 2001;30: 233–252. 10.1068/p2872 11296504

[10] Brenton J., Müller S., and Mansingh A. Discrimination of visual anticipation in skilled cricket batsmen. J Appl Sport Psychol, 2016;28: 483–488.

[11] Müller S., Fadde PJ., and Harbaugh AG. Adaptability of expert visual anticipation in baseball batting. J Sports Sci. 2016;35: 1682–1690. 10.1080/02640414.2016.1230225 27609671

[12] Müller S., Abernethy B., Reece J., Rose M., Eid M., McBean R., et al An in-situ examination of the timing of information pick-up for interception by cricket batsmen of different skill levels. Psychol Sport Exerc. 2009;10: 644–652.

[13] Müller S. and Abernethy B. Expert anticipatory skill in striking sports: A review and a model. Res Q Exerc Sport. 2012;83: 175–187. 22808703 10.1080/02701367.2012.10599848

[14] Williams AM. and Ericsson KA. Perceptual-cognitive expertise in sport: some considerations when applying the expert performance approach. Hum Mov Sci. 2005;24: 283–307. 10.1016/j.humov.2005.06.002 16095739

[15] Van der Kamp J., Rivas F., van Doorn H., and Savelsbergh G. Ventral and dorsal contributions in visual anticipation in fast ball sports. Int J Sport Psychol. 2008;39: 100–130.

[16] Müller S., Brenton J., Dempsey AR., Harbaugh AG., and Reid C. Individual differences in highly skilled visual perceptual-motor striking skill. Atten Percept Psychophys. 2015;77: 1726–1736. 10.3758/s13414-015-0876-7 25813740

[17] Bernstein NA. Coordination and regulation of movements New York: Pergamon Press; 1967.

[18] Van de Kamp C., Gawthrop P., Gollee H., Lakie M., and Loram I. D. Interfacing sensory input with motor output: does the control architecture converge to a serial process along a single channel? Front Comput Neurosci. 2013;7: 55 10.3389/fncom.2013.00055 23675342 PMC3648771

[19] Flyger N., Button C., and Rishiraj N. The science of softball: Implications for performance and injury prevention. Sports Med. 2006;36: 797–816. 10.2165/00007256-200636090-00006 16937954

[20] Adair KR. The physics of baseball 3rd ed. New York: HarperCollins; 2002.

[21] Cross R. Physics of baseball and softball New York: Springer; 2011.

[22] Müller S., Lalović A., Dempsey AR., and Rosalie SM. Pick-up of early visual information to guide kinematics and kinematics within a group of highly skilled baseball batters. Percept Mot Skills. 2014;119: 347–362. 10.2466/30.PMS.119c21z9 25244553

[23] Breunig, MM., Kriegel, H-P., Ng, RT., and Sander J. LOF: Identifying density-based local outliers. In: Proceedings of the ACM SIGMOD Conference, Dallas, TX; 2000; 93–104.

[24] Ward J. H. Jr. Hierarchical grouping to optimize an objective function. J American Stat Assoc. 1963;58: 236–244.

[25] Punj G. and David WS. Cluster analysis in marketing research: Review and suggestions for application. J Mark Res. 1983;20: 134.

[26] Ketchen D. and Shook CL. The application of cluster analysis in strategic management research: An analysis and critique. Strategic Management Journal. 1996;17: 441–458.

[27] Jain KA. and Dubes CR. Algorithms for clustering data New York: Englewood Cliffs; 1998.

[28] Katsumata H. A functional modulation for timing a movement: A coordinative structure in baseball hitting. Hum Mov Sci. 2007;26: 27–47. 10.1016/j.humov.2006.09.005 17204344

[29] Ranganathan R. and Carlton LG. Perception–action coupling and anticipatory performance in baseball batting. J Mot Behav. 2007;39: 369–380. 10.3200/JMBR.39.5.369-380 17827114

[30] Caljouw SR., van der Kamp J., and Savelsberg GJP. Timing of goal-directed hitting: Impact requirements change the information–movement coupling. Exp Brain Res. 2004;155: 135–144. 10.1007/s00221-003-1705-0 15010898

[31] Caljouw SR. and van der Kamp J. Bi-phasic hitting with constraints on impact velocity and temporal precision. Hum Mov Sci. 2005;24: 206–217. 10.1016/j.humov.2005.04.003 15964647

[32] Saltzman E. and Caplan D. A graph-dynamic perspective on coordinative structures, the role of affordance–effectivity relations in action selection, and the self-organization of complex activities. Ecol Psychol 2015;27: 300–309.

