# Supplementary figures and images for: Task constraints and stepping movement of fast-pitch softball hitting
Source: PLoS One. 2019 Feb 26;14(2):e0212997. doi: 10.1371/journal.pone.0212997 (PMC6391020; doi:10.1371/journal.pone.0212997)

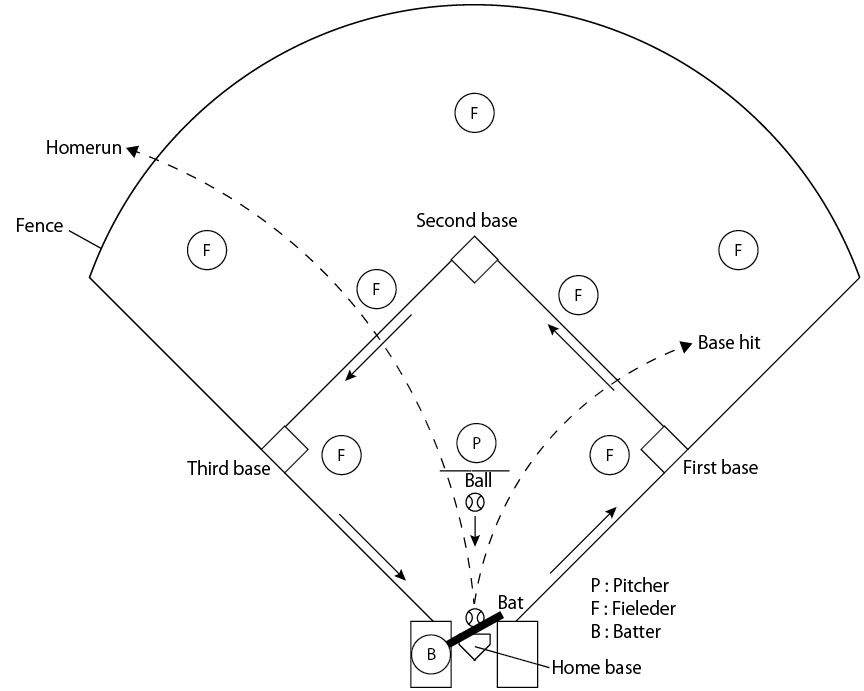

Supplement: S1 Fig — (TIFF) [file pone.0212997.s001.tiff]

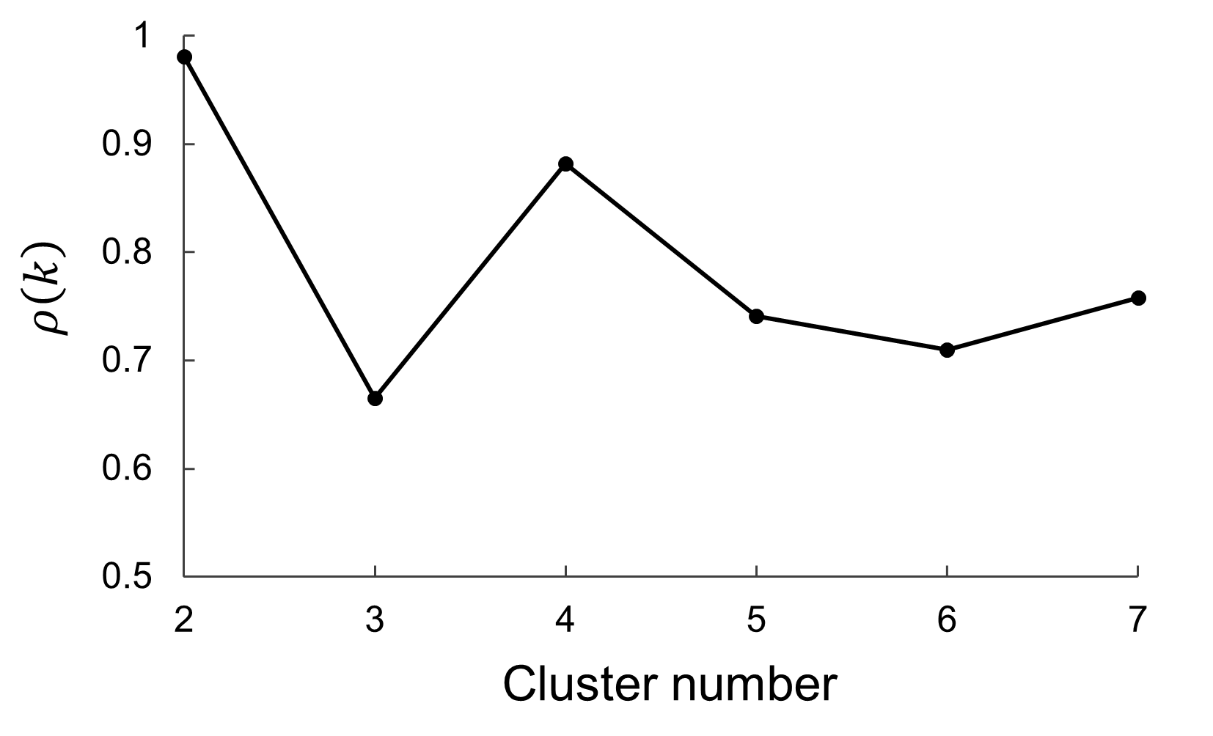

Supplement: S2 Fig — (TIF) [file pone.0212997.s002.tif]
