# Supplementary material for: Task constraints and stepping movement of fast-pitch softball hitting
Source: PLoS One. 2019 Feb 26;14(2):e0212997. doi: 10.1371/journal.pone.0212997 (PMC6391020; doi:10.1371/journal.pone.0212997)
Supplement: S1 Table — The “Measure” column indicates the team analyzed in this study (both: both teams 1 and 2 are analyzed; H: only one team is analyzed). (PDF) [file pone.0212997.s003.pdf]

Game	Team 1	Team 2	Score	Measure
1	H-A	H-B	3-0	Both
2	H-C	H-D	4-2	Both
3	H-E	H-F	1-0	Both
4	H-G	H-H	2-0	Both
5	H-I	H-J	7-0	Both
6	H-K	H-L	10-1	H-L only
7	H-M	H-K	1-0	Both
