# Supplementary material for: Task constraints and stepping movement of fast-pitch softball hitting
Source: PLoS One. 2019 Feb 26;14(2):e0212997. doi: 10.1371/journal.pone.0212997 (PMC6391020; doi:10.1371/journal.pone.0212997)
Supplement: S2 Table — The “Measure” column indicates the team analyzed in this study (both: both teams 1 and 2 are analyzed; C1, C2: only one team is analyzed). (PDF) [file pone.0212997.s004.pdf]

Game	Team 1	Team 2	Score	Measure
1	C1-A	C1-B	4-2	Both
2	C1-C	C1-D	11-0	
3	C1-E	C1-B	1-0	C1-E only
4	C1-A	C1-B	7-7	C1-C only
5	C1-E	C1-D	8-0	C1-D only
6	C1-C	C1-B	7-0	
7	C2-A	C2-B	3-1	Both
8	C2-C	C2-D	3-2	Both
9	C2-A	C2-D	11-6	
10	C2-C	C2-B	4-0	
11	C2-A	C2-C	4-4	
12	C2-B	C2-D	4-0	
