# Supplementary material for: Task constraints and stepping movement of fast-pitch softball hitting
Source: PLoS One. 2019 Feb 26;14(2):e0212997. doi: 10.1371/journal.pone.0212997 (PMC6391020; doi:10.1371/journal.pone.0212997)
Supplement: S3 Table — The “Measure” column represents the team analyzed in this study (both: both teams 1 and 2 are analyzed; L: only one team is analyzed). (PDF) [file pone.0212997.s005.pdf]

Game	Team 1	Team 2	Score	Measure
1	L-A	L-B	5-4	
2	L-C	L-D	3-1	
3	L-E	L-F	4-3	J-E only
4	L-B	L-C	4-3	Both
5	L-A	L-F	2-1	Both
6	L-E	L-D	1-0	
7	L-G	L-H	1-0	Both
8	L-F	L-B	7-2	
9	L-D	L-I	1-0	J-I only
10	L-B	L-H	2-1	
11	L-G	L-I	11-0	
12	L-D	L-F	4-3	Both
