# Supplementary material for: Task constraints and stepping movement of fast-pitch softball hitting
Source: PLoS One. 2019 Feb 26;14(2):e0212997. doi: 10.1371/journal.pone.0212997 (PMC6391020; doi:10.1371/journal.pone.0212997)
Supplement: S4 Table — (PDF) [file pone.0212997.s006.pdf]

No.	Number of pitches	Average ball travel time (s)
H1	84	0.443
H2	109	0.481
H3	98	0.409
H4	89	0.401
H5	88	0.413
H6	76	0.463
H7	78	0.427
H8	70	0.478
H9	94	0.487
H10	104	0.462
H11	98	0.495
H12	69	0.514
H13	49	0.483
Mean	88.8	0.458
