# Supplementary material for: Task constraints and stepping movement of fast-pitch softball hitting
Source: PLoS One. 2019 Feb 26;14(2):e0212997. doi: 10.1371/journal.pone.0212997 (PMC6391020; doi:10.1371/journal.pone.0212997)
Supplement: S5 Table — (PDF) [file pone.0212997.s007.pdf]

| No. | Number of pitches r | Average ball travel time (s) |
|---|---|---|
| C1 | 84 | 0.415 |
| C2 | 89 | 0.445 |
| C3 | 131 | 0.425 |
| C4 | 89 | 0.455 |
| C5 | 55 | 0.465 |
| C6 | 76 | 0.516 |
| C7 | 92 | 0.510 |
| C8 | 137 | 0.557 |
| C9 | 61 | 0.492 |
| Mean | 92.1 | 0.476 |
