# Supplementary material for: Task constraints and stepping movement of fast-pitch softball hitting
Source: PLoS One. 2019 Feb 26;14(2):e0212997. doi: 10.1371/journal.pone.0212997 (PMC6391020; doi:10.1371/journal.pone.0212997)
Supplement: S6 Table — (PDF) [file pone.0212997.s008.pdf]

No.	Number of pitches	Average ball travel time (s)
L1	102	0.406
L2	119	0.394
L3	72	0.386
L4	74	0.415
L5	105	0.439
L6	86	0.446
L7	66	0.393
L8	102	0.449
L9	93	0.410
L10	98	0.385
Mean	91.7	0.412
