# Supplementary material for: Task constraints and stepping movement of fast-pitch softball hitting
Source: PLoS One. 2019 Feb 26;14(2):e0212997. doi: 10.1371/journal.pone.0212997 (PMC6391020; doi:10.1371/journal.pone.0212997)
Supplement: S1 Supporting Information — (DOCX) [file pone.0212997.s009.docx]

**Supporting information**

**A. Rule of softball**

Figure S1 shows a softball field. “Fast-pitch” is the main type of ball throwing used in competitions such as international matches. “Slow-pitch” is a type of ball throwing that is used in recreational games; in this case, the ball must arch on its path to the batter (Flyger et al., 2006).

A softball game progresses as the two teams alternate between attacking and defense. The defense team consists of one pitcher and eight defenders (fielders). The goal in softball is to score more runs than the opposition. The attacking team’s batter can score runs by hitting the ball thrown by the opposition pitcher and running to each base in counterclockwise order. The defense team can get “outs” by catching the ball hit by the batter and throwing it to the first base before the batter reaches the first base or by catching the ball before it falls to the ground. The attacking and defense teams switch roles if the defense team obtains three outs. Both teams attack six or seven times, and the team with the highest total score wins.

A softball batter must hit the ball such that it falls between defense team players and run to the first base before being out (this is called a “base hit,” S1 Fig). Alternatively, the batter can hit the ball hard such that it goes over the fence (this is called a “homerun,” S1 Fig). Although a homerun has a larger profit than a base hit, the success rate of hitting a homerun is lower than that of a base hit because the batter needs to apply a larger force to the ball to make it go farther. These differences in successful hitting result in the redundancy of task solutions. The pitcher must throw the ball fast and accurately to prevent a hit by the batter, that is, he/she must increase the spatiotemporal difficulty of ball–bat contact (impact).

S1 Fig. Softball field.

**B. Measured games**

S1 Table. List of measured high school games. The “Measure” column indicates the team analyzed in this study (both: both teams 1 and 2 are analyzed; H: only one team is analyzed).

S2 Table. List of measured college league games. The “Measure” column indicates the team analyzed in this study (both: both teams 1 and 2 are analyzed; C1, C2: only one team is analyzed).

S3 Table. List of measured league games. The “Measure” column represents the team analyzed in this study (both: both teams 1 and 2 are analyzed; L: only one team is analyzed).

**C. Number of pitches and average ball travel time of all pitchers in each competition category**

S4 Table. Number of pitches and average ball travel time of high school pitchers.

S5 Table. Number of pitches and average ball travel time of college league pitchers.

S6 Table. Number of pitches and average ball travel time of league pitchers.

**D. Results of Jain-Dubes method**

We used the Jain-Dubes method (Jain et al., 1998) to determine the optimal number of clusters. The results of this method revealed that the optimal number of clusters, k, for the present data was three (evaluation function p(3) = 0.66).

S2 Fig. p(*k*) value with respect to cluster number as determined by the Jain–Dubes method.
